# Phenotypes in Gambling Disorder Using Sociodemographic and Clinical Clustering Analysis: An Unidentified New Subtype?

**DOI:** 10.3389/fpsyt.2019.00173

**Published:** 2019-03-29

**Authors:** Susana Jiménez-Murcia, Roser Granero, Fernando Fernández-Aranda, Randy Stinchfield, Joel Tremblay, Trevor Steward, Gemma Mestre-Bach, María Lozano-Madrid, Teresa Mena-Moreno, Núria Mallorquí-Bagué, José C. Perales, Juan F. Navas, Carles Soriano-Mas, Neus Aymamí, Mónica Gómez-Peña, Zaida Agüera, Amparo del Pino-Gutiérrez, Virginia Martín-Romera, José M. Menchón

**Affiliations:** ^1^Department of Psychiatry, University Hospital of Bellvitge-IDIBELL, Barcelona, Spain; ^2^Ciber Fisiopatologia Obesidad y Nutrición, Instituto Salud Carlos III, Barcelona, Spain; ^3^Department of Clinical Sciences, School of Medicine, University of Barcelona, Barcelona, Spain; ^4^Departament de Psicobiologia i Metodologia de les Ciències de la Salut, Universitat Autònoma de Barcelona, Barcelona, Spain; ^5^Department of Psychiatry, University of Minnesota Medical School, Minneapolis, MN, United States; ^6^Département de Psychoéducation, Université du Québec à Trois-Rivières, Trois-Rivières, QC, Canada; ^7^Department of Experimental Psychology, University of Granada, Granada, Spain; ^8^Brain, Mind and Behavior Research Center (CIMCYC), University of Granada, Granada, Spain; ^9^Ciber de Salud Mental, Instituto Salud Carlos III, Madrid, Spain; ^10^Departament d'Infermeria de Salut Pública, Salut Mental i Maternoinfantil, Escola Universitària d'Infermeria, Universitat de Barcelona, Barcelona, Spain; ^11^Department of Psychology, University of Alcalá, Alcalá de Henares, Spain

**Keywords:** gambling disorder, clustering, personality traits, psychopathology, severity

## Abstract

**Background:** Gambling disorder (GD) is a heterogeneous disorder which has clinical manifestations that vary according to variables in each individual. Considering the importance of the application of specific therapeutic interventions, it is essential to obtain clinical classifications based on differentiated phenotypes for patients diagnosed with GD.

**Objectives:** To identify gambling profiles in a large clinical sample of *n* = 2,570 patients seeking treatment for GD.

**Methods:** An agglomerative hierarchical clustering method defining a combination of the Schwarz Bayesian Information Criterion and log-likelihood was used, considering a large set of variables including sociodemographic, gambling, psychopathological, and personality measures as indicators.

**Results:** Three-mutually-exclusive groups were obtained. Cluster 1 (*n* = 908 participants, 35.5%), labeled as “high emotional distress,” included the oldest patients with the longest illness duration, the highest GD severity, and the most severe levels of psychopathology. Cluster 2 (*n* = 1,555, 60.5%), labeled as “mild emotional distress,” included patients with the lowest levels of GD severity and the lowest levels of psychopathology. Cluster 3 (*n* = 107, 4.2%), labeled as “moderate emotional distress,” included the youngest patients with the shortest illness duration, the highest level of education and moderate levels of psychopathology.

**Conclusion:** In this study, the general psychopathological state obtained the highest importance for clustering.

## Introduction

Gambling disorder (GD) is defined as a persistent and recurrent maladaptive pattern of gambling behavior associated with impaired functioning in the personal, social, and occupational spheres of one's life (1). Across the globe, GD affects 0.7–6.5% of adults during their lifetime (2). Evidence points to GD as being more likely to occur among males, singles or divorcees, unemployed individuals or with a low income, and with a lower level of education (3). Moreover, GD presents high comorbidity with other mental disorders, mainly substance or alcohol dependence (4) and personality disorders (5).

From an etiological point of view, GD is a multi-causal disorder involving psychosocial and neurobiological risk factors. The interaction between these factors and the specific implication of each can aid in discerning the phenotypes of GD patients. One of the psychosocial risk factors of greatest interest has been psychological or emotional distress, which is related to high levels of general psychopathology as measured by the Symptom Checklist-Revised (SCL-90-R) (6, 7). In this sense, Blaszczynski and Nower (8) pointed out the existence of a subtype of individuals with GD characterized by high emotional vulnerability. These authors hypothesized that emotional vulnerability is a result of different psychosocial factors, such as childhood disturbances, certain personality traits, mood alterations (depression and anxiety), poor coping skills, and substance use. In this context, gambling is viewed as a means of relieving aversive affective states. Posterior evidence has concluded that these “emotionally vulnerable” gamblers exhibit higher psychopathological and gambling severity than other subtypes of gamblers, as well as a higher prevalence of substance abuse and an earlier onset of gambling problems (9–12).

On the one hand, problematic or pathological gambling behavior has frequently been associated with certain types of gambling activities. That is, not all gambling activities seem to be addictive to the same degree. Until recently, slot machines were considered the most potentially addictive type of gambling because of their technical and situational characteristics (13). However, the emergence and proliferation of online gambling in the last decade has radically modified the characteristics of gambling activities, by which gambling settings have been shifted to home and work environments (14). Online gambling is considered potentially more addictive because of its unique features, such as anonymity, betting speed, accessibility, attractive design, and marketing, and low cost (15, 16). Indeed, recent evidence has displayed that online gambling increases the likelihood of being a problematic gambler when compared with offline gambling (16). Although online gamblers seem to be an heterogeneous group, their profile is often the following: male, young, single, high levels of education, good economic income, varied gambling preferences, greater severity of gambling behavior (in terms of spending higher amounts), as well as higher rates of alcohol and substance use (17–19). Likewise, it can be expected that specific gambling activities have a higher representation in certain subtypes of gamblers, for instance, online gambling may be more frequent in those subtypes composed of younger gamblers with higher education, with better incomes and the possibility of betting larger amounts of money.

Beyond these results, the question is whether it is possible to formulate the hypothesis that emotional alterations and the type of preferred gambling could explain the existence of more specific subtypes. To the best of our knowledge, no studies have explored the last question in large clinical samples made up of consecutive patients who have requested specific treatment for gambling problems. For that reason, the objective of this study is to explore the existence of distinct GD phenotypes, in a large sample of patients seeking treatment for GD, through clustering analysis, using a wide set of variables including sociodemographic, gambling-related factors, general psychopathological state and personality traits as indicators for the grouping. Based on the available evidence in literature, we hypothesized that different empirical clusters would emerge based on emotional distress. Due to the dramatic change in relation to the proliferation of different types of gambling in this last decade (specifically, the popularization of online gambling) and the change in profiles of people affected by GD, our secondary hypothesis was that one of these clusters would overlap with the emerging profile of the online gambler.

## Materials and Methods

### Participants

The study sample included *n* = 2,570 patients treated at the Gambling Disorder Unit at the Department of Psychiatry of Bellvitge University Hospital, in Barcelona, Spain. This public hospital is certified as a tertiary care center for the treatment of psychologically addictive behaviors and it oversees the treatment of complex cases. The catchment area of the hospital includes over two million people in the metropolitan area of Barcelona.

All patients that met diagnostic criteria for GD, between the ages of 18 and 75, were selected for this study. Patients were recruited between January 2005 and November 2015. Exclusion criteria included having an intellectual disability or severe mental disorders (schizophrenia or other psychotic disorders, bipolar disorder, etc.), or meeting criteria for another behavioral addiction apart from GD.

Most participants were men (*n* = 2,365, 92.0%), born in Spain (*n* = 2,405, 93.6%), were married or had a partner (*n* = 1,324, 51.5%) and were employed (*n* = 1,525, 59.3%). The mean age for the total sample was 41.7 years old (SD = 12.8), the mean age of GD onset was 36.8 years old (SD = 12.9) and the mean duration of GD was 14.0 years (SD = 10.0). The first columns of Table 1 include a description of the study sample.

**Table 1:** Cluster composition and comparison between groups.

**Categorical variables**		**Total**		**Cluster 1**		**Cluster 2**		**Cluster 3**		**Cluster**	**Pairwise comparisons:** ***p***		
		***n*** **=2,570**		***n*** **=** **908; 35.3%**		***n*** **=** **1,555; 60.5%**		***n*** **=** **107; 4.2%**					
		***n***	**%**	***n***	**%**	***n***	**%**	***n***	**%**	***p*-value**	**C1-C2**	**C1-C3**	**C2-C3**
Origin	Spanish	2,405	93.6%	836	92.1%	1483	95.4%	86	80.4%	**<0.001**	**0.001**	**<0.001**	**<0.001**
	Immigrant	165	6.4%	72	7.9%	72	4.6%	21	19.6%				
Sex	Men	2,365	92.0%	782	86.1%	1495	96.1%	88	82.2%	**<0.001**	**<0.001**	0.278	**<0.001**
	Women	205	8.0%	126	13.9%	60	3.9%	19	17.8%				
Education	Primary	1443	56.1%	553	60.9%	873	56.1%	17	15.9%	**<0.001**	**0.039**	**<0.001**	**<0.001**
	Secondary	973	37.9%	319	35.1%	597	38.4%	57	53.3%				
	University	154	6.0%	36	4.0%	85	5.5%	33	30.8%				
Civil status	Single	883	34.4%	355	39.1%	486	31.3%	42	39.3%	**<0.001**	**<0.001**	0.522	**<0.001**
	Married	1,324	51.5%	395	43.5%	887	57.0%	42	39.3%				
	Divorced-widow	363	14.1%	158	17.4%	182	11.7%	23	21.5%				
Employed	Yes	1525	59.3%	445	49.0%	993	63.9%	87	81.3%	**<0.001**	**<0.001**	**<0.001**	**<0.001**
Smoking use-abuse	Yes	1,668	64.9%	605	66.6%	1025	65.9%	38	35.5%	**<0.001**	0.718	**<0.001**	**<0.001**
Alcohol use-abuse	Yes	385	15.0%	155	17.1%	228	14.7%	2	1.9%	**<0.001**	0.112	**<0.001**	**<0.001**
Drugs use-abuse	Yes	252	9.8%	125	13.8%	121	7.8%	6	5.6%	**<0.001**	**<0.001**	**0.017**	0.413
**Quantitative variables**	**α**	**Mean**	**SD**	**Mean**	**SD**	**Mean**	**SD**	**Mean**	**SD**				
Age (years-old)		41.73	12.81	42.43	12.45	41.49	13.08	39.30	11.46	**0.029**	0.079	**0.017**	0.087
Onset (years-old)		36.76	12.88	36.65	12.90	36.92	12.98	35.36	11.25	0.457	0.611	0.330	0.227
Duration (years)		13.99	9.97	14.86	9.79	13.62	10.03	11.96	10.14	**0.001**	**0.003**	**0.004**	0.095
**GD SEVERITY MEASURES**													
Mean bets-episode		129.4	629.7	105.7	196.1	88.8	143.1	920.1	2884.5	**<0.001**	0.507	**<0.001**	**<0.001**
Cumulate debts		10,858	77219	8551	23509	6431	17847	94759	357317	**<0.001**	0.500	**<0.001**	**<0.001**
# gambling activities		1.02	0.22	1.01	0.09	1.00	0.05	1.42	0.92	**<0.001**	0.454	**<0.001**	**<0.001**
SOGS-total score	0.757	10.82	2.96	12.09	2.64	10.05	2.86	11.17	3.15	**<0.001**	**<0.001**	**0.001**	**<0.001**
DSM-5 total criteria	0.807	6.78	1.99	7.70	1.46	6.24	2.04	6.83	2.28	**<0.001**	**<0.001**	**<0.001**	**0.002**
**SCL-90R SCALES**													
Somatization	0.909	0.94	0.83	1.69	0.81	0.51	0.46	0.83	0.63	**<0.001**	**<0.001**	**<0.001**	**<0.001**
Obsessive-com.	0.881	1.12	0.83	1.92	0.72	0.65	0.47	1.18	0.63	**<0.001**	**<0.001**	**<0.001**	**<0.001**
Interp. sensitivity	0.872	1.02	0.84	1.87	0.73	0.54	0.44	0.98	0.53	**<0.001**	**<0.001**	**<0.001**	**<0.001**
Depressive	0.910	1.48	0.92	2.39	0.67	0.94	0.57	1.57	0.71	**<0.001**	**<0.001**	**<0.001**	**<0.001**
Anxiety	0.896	1.01	0.83	1.84	0.74	0.52	0.39	0.97	0.55	**<0.001**	**<0.001**	**<0.001**	**<0.001**
Hostility	0.852	0.91	0.84	1.62	0.89	0.49	0.46	0.89	0.71	**<0.001**	**<0.001**	**<0.001**	**<0.001**
Phobic anxiety	0.821	0.48	0.69	1.04	0.84	0.17	0.26	0.32	0.49	**<0.001**	**<0.001**	**<0.001**	**0.006**
Paranoia	0.782	0.90	0.79	1.60	0.77	0.50	0.45	0.86	0.59	**<0.001**	**<0.001**	**<0.001**	**<0.001**
Psychotic	0.858	0.89	0.77	1.63	0.71	0.46	0.38	0.83	0.56	**<0.001**	**<0.001**	**<0.001**	**<0.001**
GSI index	0.981	1.04	0.72	1.82	0.55	0.59	0.32	1.03	0.46	**<0.001**	**<0.001**	**<0.001**	**<0.001**
PST index	0.981	45.90	21.69	67.72	11.84	33.00	15.32	48.35	15.30	**<0.001**	**<0.001**	**<0.001**	**<0.001**
PSDI index	0.981	1.88	0.61	2.41	0.52	1.58	0.43	1.87	0.45	**<0.001**	**<0.001**	**<0.001**	**<0.001**
**TCI-R SCALES**													
Novelty seeking	0.702	108.89	14.40	111.80	13.95	106.94	14.31	112.58	14.66	**<0.001**	**<0.001**	0.592	**<0.001**
Harm avoidance	0.806	101.09	17.08	110.25	15.96	95.70	15.34	101.78	17.22	**<0.001**	**<0.001**	**<0.001**	**<0.001**
Reward dependence	0.763	98.54	14.85	95.48	15.25	100.33	14.38	98.46	13.92	**<0.001**	**<0.001**	**0.047**	0.202
Persistence	0.862	108.46	20.03	107.05	20.41	109.44	19.70	106.21	20.69	**0.008**	**0.004**	0.679	0.105
Self-directedness	0.846	126.93	21.16	112.13	16.73	135.68	18.60	125.37	19.64	**<0.001**	**<0.001**	**<0.001**	**<0.001**
Cooperativeness	0.802	130.43	16.43	123.28	16.78	134.52	14.78	131.74	15.75	**<0.001**	**<0.001**	**<0.001**	0.074
Self-transcendence	0.830	64.15	15.38	69.04	15.60	61.46	14.55	61.60	15.17	**<0.001**	**<0.001**	**<0.001**	0.927

*SD, standard deviation; α, Chronbach's alpha in the sample; Bold, significant comparison (0.05 level)*.

*p-value includes Finner's correction for multiple statistical comparison*.

### Instruments

#### GD Diagnosis and Severity

##### South Oaks Gambling Screen (SOGS) (20)

This is a self-report 20-item screening questionnaire that discriminates between probable pathological, problem and non-problem gamblers. The Spanish validation used in this work showed excellent internal consistency (α = 0.94) and test-retest reliability (*r* = 0.98) (21).

##### Diagnostic Questionnaire for Pathological Gambling According to DSM-IV Criteria (22). Spanish Adaptation by Jiménez-Murcia et al. (23).

This 19-item questionnaire assesses the DSM-IV diagnostic criteria for pathological gambling. It should be noted that with the release of the DSM-5 (1), the term pathological gambling was replaced with GD. Internal consistency of this questionnaire ranged between α = 0.81 for the general population and α = 0.77 for gambling treatment samples. Convergent validity with the SOGS scores was high: *r* = 0.77 for the general population and *r* = 0.75 for gambling treatment groups (22). Due to the fact that this questionnaire includes the 8th criterion exploring the presence of illegal acts related to GD, in this study, all patient diagnoses were reassessed and recodified *post hoc* to avoid the confounding effect of increased GD severity, in patients with a criminal history. Therefore, only patients who met DSM-5 criteria for GD were included in our analysis.

#### Personality and Psychopathological Status

##### Temperament and Character Inventory-Revised (TCI-R) (24)

This is a reliable and valid 240-item questionnaire that measures seven personality dimensions: four temperament (novelty seeking, harm avoidance, reward dependence and persistence) and three character dimensions (self-directedness, cooperativeness and self-transcendence). Temperament refers to automatic emotional responses to experiences that are moderately heritable and stable throughout life. In contrast, character refers to self-concepts and individual differences in goals and values. Character is moderately influenced by insight and learning. All items are measured on a 5-point Likert-type scale. A validated Spanish version was used (25). The scales in the Spanish revised version showed adequate internal consistency (Cronbach's α mean value of 0.87). In the study at hand, consistency indices ranged from good (α = 0.70 for novelty seeking) to very good (α = 0.84 for persistence and self-transcendence).

##### Symptom Checklist-Revised (SCL-90-R) (26)

This questionnaire is a measure of emotional distress and evaluates a broad range of psychological problems and psychopathological symptoms. This questionnaire contains 90 items and assesses nine primary symptom dimensions: somatization, obsession-compulsion, interpersonal sensitivity, depression, anxiety, hostility, phobic anxiety, paranoid ideation, and psychoticism. It also includes three global indices: (1) a global severity index (GSI), designed to measure overall psychological distress; (2) a positive symptom distress index (PSDI), to measure the intensity of the symptoms; and (3) a positive symptom total (PST), which reflects self-reported symptoms. A validated Spanish version was used (27). The Spanish validation scale obtained good psychometrical indexes, with a mean internal consistency of 0.75 (Cronbach's alpha). In the study sample, consistency indices were in the very good (α = 0.83 for hostility) to excellent range (α = 0.98 for the global indexes), with exception of paranoia (α = 0.74, good).

#### Other Sociodemographic and Clinical Variables

Additional demographic, clinical, and social/family variables related to gambling were measured using a semi-structured, face-to-face clinical interview described elsewhere (28). Some of the GD variables covered included the age of GD onset, the mean and maximum amount of monetary spending in gambling episodes, and accumulated gambling-related debts. Moreover, GD severity was assessed according to five different variables: total number of DSM-5 criteria, SOGS-total score, number of gambling activities, mean bets per gambling episode, and cumulative debts.

Table 1 contains the α-values obtained in the sample for all the scales in this work. These coefficients ranged between good to excellent.

### Procedure

The present study was carried out in accordance with the latest version of the Declaration of Helsinki. The University Hospital of Bellvitge Ethics Committee of Clinical Research approved the study (ref. 307/06), and signed informed consent was obtained from all participants. Experienced psychologists and psychiatrists with more than 15 years of clinical experience in the field of addictive disorders conducted the two face-to-face clinical interviews. All the measures used in this study correspond to the assessment carried out prior to the beginning of treatment.

### Statistical Analysis

Statistical analysis was carried out with SPSS 20 for Windows. Empirical clusters were explored through the TwoStep-Clustering Component, entering origin (Spanish vs. immigrant), sex, education level, civil status, employment status, age, age of GD onset, duration of the gambling problem, GD severity and gambling related variables (SOGS-total score, number of gambling activities, mean bets per gambling episode and cumulative debts), comorbid psychopathological state (SCL-90R scales), substances use-abuse (tobacco, alcohol and other drugs) and personality traits (TCI-R scales) as indicators. The TwoStep-Clustering system constitutes a scalable algorithm designed to handle large datasets including both continuous and categorical variables. During the first step, subjects are pre-clustered into many small sub-clusters according to a sequential clustering approach and during the second step the resulting sub-clusters are considered as inputs and grouped into the desired number of clusters according to the agglomerative hierarchical clustering method. By default, the TwoStep algorithm uses a combination of the Schwarz Bayesian Information Criterion and log-likelihood distance in determining the final number of clusters, choosing a solution with a reasonably large ratio of Schwarz Bayesian Information Criterion changes and a large ratio of distance measures. The log-likelihood measure is computed by using the normal density for continuous variables and the multinomial probability mass function for categorical variables. In this study, we compared the automatic number of clusters selected by the TwoStep Clustering procedure as finalistic candidate solutions, and two additional models: the auto-determined number of clusters minus one, and the auto-determined number plus one. The final chosen model was based on (29) criteria: (a) the highest cohesion and separation index; (b) adequate number of individuals in each group (to allow for meaningful comparisons); and (c) the best clinical interpretability.

The comparison between the derived empirical clusters for the study variables (sociodemographic, clinical, and personality measures) was carried out with chi-square tests (χ^2^) for categorical factors and analysis of variance (ANOVA) for quantitative measures. Finner's correction (a familywise error rate stepwise procedure which offers more powerful test than the classical Bonferroni correction) controlled for inflation in Type-I error due to multiple statistical comparisons (30).

## Results

### Cluster Composition and Comparison Between Groups

The three-cluster solution was selected as being the most optimal in the sample of *n* = 2,570 GD patients who met inclusion criteria. This structure was auto-determined as the most appropriate by the TwoStep-Clustering procedure. The Silhouette's index [a validity measure of consistency within clusters, which can be interpreted as the level of cohesion/separation for the empirical derived groups; (31)] was moderate (average Silhouette = 0.3, suggesting reasonable evidence of cluster structure in the data). The comparison between the largest cluster size (*n* = 1,555, 60.5%) and the smallest (*n* = 107, 4.2%) yielded a ratio of 14.53.

**Figure 1:**
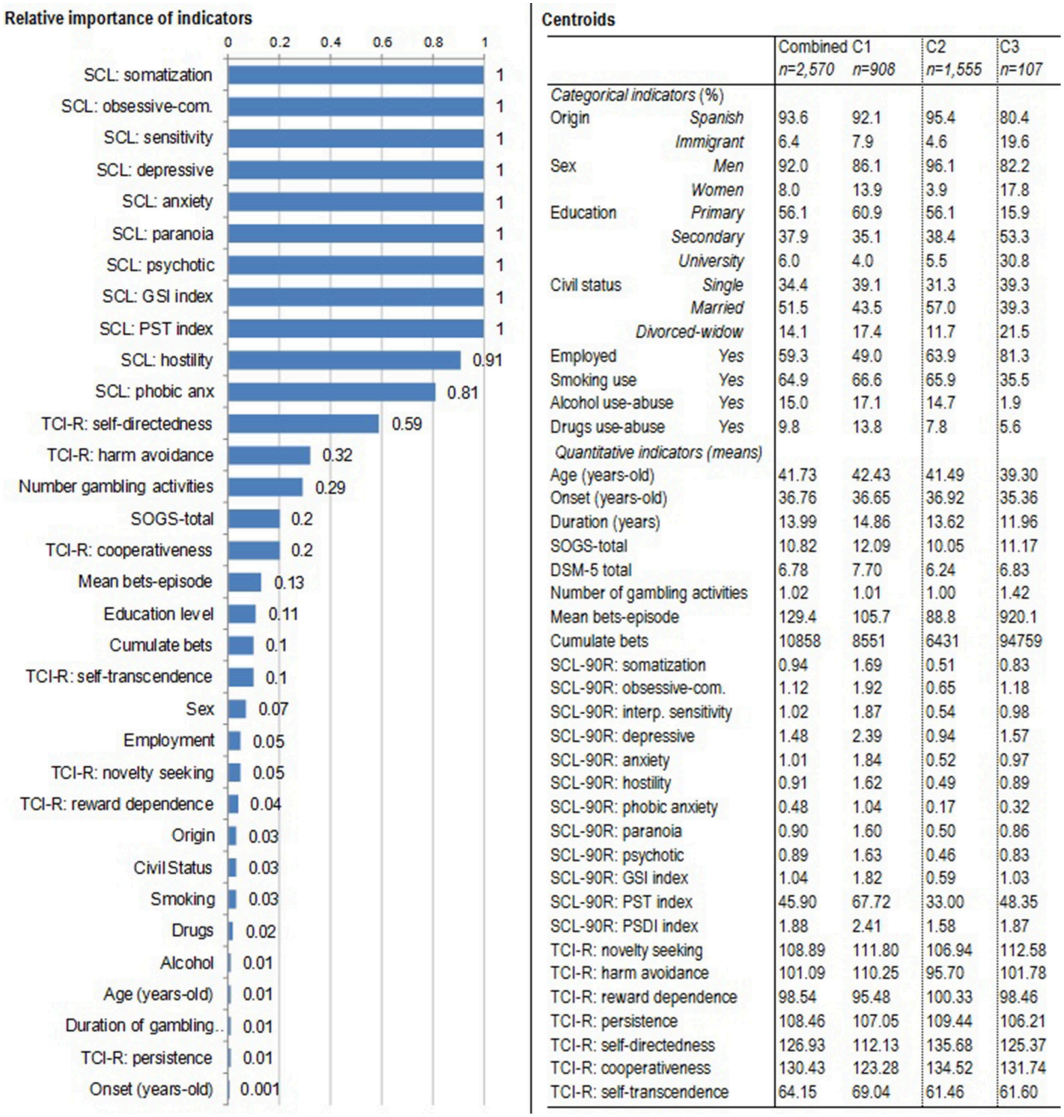
Clustering summary: relative importance of each indicator and centroids.

Figure 1 summarizes the composition of the final solution for the three-cluster model. The first panel contains the bar-graph with the relative importance of each indicator in the clustering, which reports how well each variable can differentiate the different derived clusters (the higher the importance of the measure, the less likely it is that the variation for the variable between clusters is due to chance and the more likely it is due to underlying differences). In this study, the general psychopathological state registered on the SCL-90-R questionnaire obtained the highest relative importance for clustering, followed by the TCI-R self-directedness and harm avoidance dimensions, and the GD severity measures (number of gambling activities, and SOGS-total score). The poorest relevance for clustering was obtained for the sample origin (Spanish vs. immigrant), civil status, substances use-abuse, chronological age, illness duration, TCI-R persistence scores, and age of GD onset. The table included in the right panel of Figure 1 contains the centroids for the indicators in the clustering (means for the quantitative variables and the percentage distribution for categorical variables), which summarizes the clusters patterns for this set of variables.

Cluster 1 contained 908 patients (35.5% of the sample) and it was characterized by featuring the oldest patients with the longest duration of GD, the highest GD severity, the most severe levels of general psychopathology or emotional distress (highest mean scores on the SCL-90R questionnaire), the highest levels in the personality traits harm avoidance and self-transcendence and the lowest levels in the personality domains of self-directedness and cooperativeness. This cluster has been labeled as “high emotional distress.”

Cluster 2, the largest empirical group with 1,555 patients (60.5% of the sample), was characterized by including the highest proportion of men, the lowest GD severity, the best psychopathological state (lowest means on the SCL-90R scales), the lowest levels in the personality traits novelty seeking and harm avoidance, and the highest mean scores in the personality scales reward dependence, persistence, self-directedness, and cooperativeness. This cluster has been labeled as “mild emotional distress.”

Cluster 3, with only 107 patients (4.2%), included the youngest participants with the lowest GD duration, the highest levels of education, the lowest prevalence of substance use-abuse, the most severe economical consequences related to the GD (mean bets per episode and cumulate debts due to gambling), and moderate levels in psychopathological state. This cluster has been labeled as “moderate emotional distress.”

### Distribution of the Empirical Clusters During the Period of Data Recruitment

**Figure 2:**
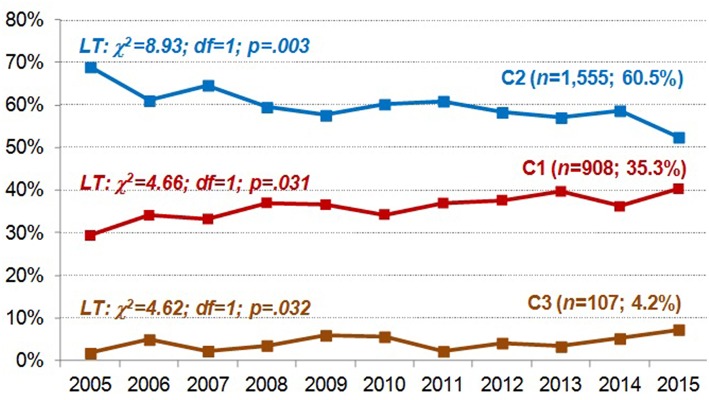
Distribution of the prevalence of each cluster during the data recruitment. LT, linear trend.

The line-chart in the Figure 2 shows the prevalence of each cluster in the study sample during the data recruitment period. As a whole, the most prevalent cluster during the time of data recruitment was cluster 2 (“mild emotional distress”), followed by cluster 1 (“high emotional distress”). A positive significant linear trend for clusters 1 (its presence increased from 29.4% in 2005 to 40.3% in 2015) and 3 (increases ranged from 1.7% in 2005 to 7.3% in 2015) was found, while cluster 2 obtained a negative significant linear trend (prevalence decreased from 68.9% in 2005 to 52.4% in 2015).

### Comparisons Between Clusters

The last columns of Table 1 contain the results of the comparison between the groups for the variables analyzed in the study and defined as indicators in the clustering procedure.

Table 2 includes the gambling preferences and the comparisons between clusters. The preferred gambling activity was slot machines for clusters 1 and 2, while cluster 3 included a higher proportion of participants reporting online gambling, but also bingo, casinos, and land-based sport betting as their main preferences.

**Table 2:** Comparison between clusters based on main gambling preference.

	**Total;** ***n*** **=** **2,570**		**C1;** ***n*** **=** **908**		**C2;** ***n*** **=** **1,555**		**C3;** ***n*** **=** **107**		**Cluste*r***	**Pairwise comparison:** ***p***		
	***n***	**%**	***n***	**%**	***n***	**%**	***n***	**%**	***p***	**C1-C2**	**C1-C3**	**C2-C3**
**PRIMARY ACTIVITY**												
Slot machines	2036	79.2%	739	81.4%	1257	80.8%	40	37.4%	**<0.001**	0.736	**<0.001**	**<0.001**
Bingo	274	10.7%	139	15.3%	113	7.3%	22	20.6%	**<0.001**	**<0.001**	0.160	**<0.001**
Lotteries	236	9.2%	95	10.5%	126	8.1%	15	14.0%	**0.031**	**0.048**	0.263	**0.034**
Casinos	189	7.4%	78	8.6%	89	5.7%	22	20.6%	**<0.001**	**0.006**	**<0.001**	**<0.001**
Cards	92	3.6%	44	4.8%	38	2.4%	10	9.3%	**<0.001**	**0.001**	**0.050**	**<0.001**
Offline betting	103	4.0%	36	4.0%	51	3.3%	16	15.0%	**<0.001**	0.374	**<0.001**	**<0.001**
Online gambling	159	6.2%	44	4.8%	85	5.5%	30	28.0%	**<0.001**	0.505	**<0.001**	**<0.001**
Other	68	2.6%	16	1.8%	38	2.4%	14	13.1%	**<0.001**	0.265	**<0.001**	**<0.001**
**SECONDARY ACTIVITY**												
Slot machines	68	2.6%	22	2.4%	39	2.5%	7	6.5%	**0.037**	0.896	**0.016**	**0.014**
Bingo	155	6.0%	67	7.4%	84	5.4%	4	3.7%	0.083	**0.048**	0.163	0.457
Lotteries	327	12.7%	132	14.5%	186	12.0%	9	8.4%	0.071	0.066	0.083	0.270
Casinos	57	2.2%	25	2.8%	29	1.9%	3	2.8%	0.323	0.146	0.976	0.494
Cards	69	2.7%	23	2.5%	44	2.8%	2	1.9%	0.788	0.663	0.675	0.558
Offline betting	41	1.6%	13	1.4%	26	1.7%	2	1.9%	0.876	0.645	0.723	0.878
Online gambling	19	0.7%	6	0.7%	11	0.7%	2	1.9%	0.375	0.893	0.181	0.187
Other	18	0.7%	9	1.0%	7	0.5%	2	1.9%	0.100	0.107	0.407	0.053

*Bold, significant comparison (0.05 level); Bonferroni-Finner's correction for multiple statistical comparison*.

## Discussion

The aim of this study was to explore the existence of distinct GD phenotypes in a large sample of patients seeking treatment for GD through clustering analysis, using a wide set of variables, including sociodemographic, gambling-related factors, general psychopathological state, and personality traits as indicators. We expected that different empirical clusters would emerge based on emotional distress or psychopathological state, measured using SCL-90R questionnaire.

In fact, in keeping with models proposing emotion-regulation processes to be strongly related to individual differences among GD patients (8, 32), the most relevant variable for the clustering analysis was emotional distress (SCL-90-R); followed by personality traits (TCI-R), and GD severity measures (number of gambling activities and SOGS-total score). Thus, three empirical clusters emerged defining psychopathological status as their main indicator: (a) Cluster 1 composed of more dysfunctional patients who reported high emotional distress; (b) Cluster 2 grouped patients with low distress levels; (c) Cluster 3 characterized by mainly young patients with medium distress levels.

From a clinical perspective, these findings could be understood taking into account that certain personality traits or domains could be associated with a greater or lesser degree of psychopathology or emotional distress, as shown in previous research (33–37). Specifically, according to the results, the personality trait most closely associated with psychopathology or emotional distress was harm avoidance. All the mentioned personality and emotional vulnerability characteristics make Cluster 1 especially complex from a therapeutic point of view, given that these patients present greater gambling severity, more vulnerability to stressful situations and specific personality characteristics that hinder their ability to cope with adverse situations. These personality traits are characterized by a greater tendency to anxiety, worry and insecurity, isolation, and disconnection from the environment, poor decision-making skills and planning skills (38). In this profile, therefore, gambling could be a dysfunctional habit acquired and maintained over time as a mechanism to avoid problems and difficulties in emotion regulation. Thus, it could represent a group of patients more inclined to present poor response to treatment, as various studies have previously shown (39–41). As such, they may require treatment programs that incorporate techniques to improve these aspects, favoring a better response to therapy (8).

Another interesting aspect to highlight from these results is that patients with the highest emotional distress and severity were the oldest patients with the longest GD duration, most of them included in the Cluster 1. This result would be in agreement with previous studies that have focused on exploring the clinical differences of patients diagnosed with GD, depending on age (42–44). The findings of these studies suggest that age is a strong moderating variable in the evolution of the disorder and that it has a relevant impact on the emotional aspects associated with gambling. In this sense, in the elderly, anxious-depressive symptoms would be related to medical illnesses and health problems, but also to stressful life situations that appear at this stage of life (e.g., retirement, reduced purchasing power, loneliness, loss of family or friends, etc.) (35, 45, 46).

Patients from our study also displayed different preferences in gambling activities, depending on the cluster that they were formed part of. Most of patients included in Cluster 1 and Cluster 2 preferred slot machines. In contrast, Cluster 3 was mainly composed of patients with a higher variety of gambling preferences, even though the most frequent gambling activity was again slot machines, these were nearly followed by online gambling. These results are in agreement with other studies having observed that online gamblers usually also engaged in land-based gambling, combining both modalities (47). Literature supports that online gambling is mainly practiced by young men (17–19), according to our results where Cluster 3 was composed of the youngest patients who also prefer new forms of gambling activities such as online gambling. The increase in the use of online gambling may be because of features that make it more convenient and accessible (15, 16), especially for young people. Relatedly, the rest of features of patients included in Cluster 3 also coincide with profile of online gamblers, being male, high levels of education, employed with good incomes, betting larger amounts of money, as well as diverse gambling preferences (17–19).

In more general terms, Cluster 3 seems to largely overlap with a subtype of gambler at least partially identified in previous research. On the one hand, this cluster is likely be responsible for the pattern of correlations between different types of high arousal, large-stake games (bets, casino games, skill-based games) categorized as Type I gambling in Navas et al. (48). On the other hand, GD patients with this profile (even after controlling for age and other potential confounders) are more likely to present high sensation- or novelty-seeking scores and stronger gambling-related cognitive distortions. At the same time, and somewhat counterintuitively, in these patients, general self-regulatory, and executive functioning, as well as high-order cognitive processing, tend to be well preserved (32, 49, 50). Integrity of high-order cognitive and regulatory processes is fully compatible with the fact that Cluster 3 GD patients present a higher level of education, higher self-directedness scores, and a lower risk of suffering from a comorbid addictive disorder. In spite of these seemingly “favorable” sociodemographic and neurocognitive features, gambling severity is by no means reduced in this profile. Actually, the variable cumulated amount of debts exceeded 94,000 euros in Cluster 3, while in the other groups, it did not reach 10,000 euros. Different studies point out that, in online gambling, spending money is much easier and faster; therefore, debts are acquired in a shorter period of time (14, 47).

Online gambling has brought about the appearance of a new GD phenotype, designated as Cluster 3 in our study. Although it includes fewer patients (only 4.2% of the total sample), Cluster 3 represents a completely new and differentiated type of GD patient, with respect to the previous subgroup identification studies (8, 10–12). We can postulate, seeing how the online gambling market is increasing and attracting young people, that this group will be more frequent in the near future. Therefore, it is necessary to identify indicators of good or bad response to treatment to be able to manage this new and growing phenotype. More specifically, novelty- and sensation-seeking—seemingly elevated in Cluster 3—have been observed to be among the best predictors of low treatment adherence and compliance (51–53). This finding is not only relevant to tailoring treatment plans, but it could also indicate that the low percentage of Cluster 3 patients in clinical sample could be partially due to the fact that a some potential patients with such a profile are less prone to seek or remain in treatment.

Finally, the progression of the clusters throughout time is information of clinical and therapeutic interest. It can be seen how Cluster 2 decreased significantly whereas Clusters 1 and 3 have increased rates. It could be possible that we are at a crucial point in terms of a change in the clinical profiles of GD individuals, which may require the development of new theoretical models and therapeutic strategies that adapt to these changes.

### Limitations

The present study has the potential to improve current knowledge on GD subtypes and provides relevant information by describing three different clusters depending on different emotional alterations. However, this research has some limitations that need to be considered. First of all, our sample was principally made of males referred to a specialized unit that usually deals with severe cases of GD. For this reason, our results have to be taken with caution before being generalized to the general population. Future studies should be conducted to explore if the described subtypes are applicable to both sexes and include samples from other centers. Second, this is a cross-sectional study; therefore, no causality can be driven from the results. Future studies should test if the observed clusters could also predict treatment outcome.

## Conclusion

Cluster formation was carried out mainly taking into account the psychopathological state or emotional distress, using the SCL-90-R. This variable allowed for differentiation of homogeneous groups or subtypes within the spectrum of the disorder. In agreement with Chamberlain (54), the identification of differentiated subtypes can contribute to a better understanding of GD from a clinical perspective. Characterizing phenotypes and endophenotypes will favor the design and implementation of increasingly specific interventions that improve response to treatment.

## Author Contributions

SJ-M, RG, and FF-A designed the experiment and analyzed the data. SJ-M, RG, FF-A, TS, GM-B, NM-B, NA, MG-P, and ZA conducted the experiment and wrote a first draft of the manuscript. TS, GM-B, RS, JT, ML-M, TM-M, JP, JN, CS-M, AdP-G, VM-R, and JM further reviewed and modified the manuscript.

### Conflict of Interest Statement

The authors declare that the research was conducted in the absence of any commercial or financial relationships that could be construed as a potential conflict of interest.
