# Corrigendum: Phenotypes in Gambling Disorder Using Sociodemographic and Clinical Clustering Analysis: An Unidentified New Subtype?

**DOI:** 10.3389/fpsyt.2019.00358

**Published:** 2019-05-28

**Authors:** Susana Jiménez-Murcia, Roser Granero, Fernando Fernández-Aranda, Randy Stinchfield, Joel Tremblay, Trevor Steward, Gemma Mestre-Bach, María Lozano-Madrid, Teresa Mena-Moreno, Núria Mallorquí-Bagué, José C. Perales, Juan F. Navas, Carles Soriano-Mas, Neus Aymamí, Mónica Gómez-Peña, Zaida Agüera, Amparo del Pino-Gutiérrez, Virginia Martín-Romera, José M. Menchón

**Affiliations:** ^1^Department of Psychiatry, University Hospital of Bellvitge-IDIBELL, Barcelona, Spain; ^2^Ciber Fisiopatologia Obesidad y Nutrición, Instituto Salud Carlos III, Barcelona, Spain; ^3^Department of Clinical Sciences, School of Medicine, University of Barcelona, Barcelona, Spain; ^4^Departament de Psicobiologia i Metodologia de les Ciències de la Salut, Universitat Autònoma de Barcelona, Barcelona, Spain; ^5^Department of Psychiatry, University of Minnesota Medical School, Minneapolis, MN, United States; ^6^Département de Psychoéducation, Université du Québec à Trois-Rivières, Trois-Rivières, QC, Canada; ^7^Department of Experimental Psychology, University of Granada, Granada, Spain; ^8^Brain, Mind and Behavior Research Center (CIMCYC), University of Granada, Granada, Spain; ^9^Ciber de Salud Mental, Instituto Salud Carlos III, Madrid, Spain; ^10^Departament d’Infermeria de Salut Pública, Salut Mental i Maternoinfantil, Escola Universitària d’Infermeria, Universitat de Barcelona, Barcelona, Spain; ^11^Department of Psychology, University of Alcalá, Alcalá de Henares, Spain

**Keywords:** gambling disorder, clustering, personality traits, psychopathology, severity

In the original article, the reference for “(48)” was incorrectly written as “48. Navas JF, Billieux J, Perandrés-Gómez A, López-Torrecillas F, Cándido A, Penelo E. Impulsivity traits and gambling cognitions associated with gambling preferences and clinical status. Int Gambl Stud. (2017) 17:102–24. doi: 10.1080/14459795.2016.1275739.” It should be “48. Navas JF, Billieux J, Perandrés-Gómez A, López-Torrecillas F, Cándido A, Perales JC. Impulsivity traits and gambling cognitions associated with gambling preferences and clinical status. Int Gambl Stud. (2017) 17:102–24. doi: 10.1080/14459795.2016.1275739.”

Additionally, the reference for “(49)” was incorrectly written as “49. Penelo E, Navas JF, Ruiz de Lara CM, Maldonado A, Catena A. Causal learning in gambling disorder: beyond the illusion of control. J Gambl Stud. (2017) 33:705–17. doi: 10.1007/s10899-016-9634-6.” It should be “49. Perales JC, Navas JF, Ruiz de Lara CM, Maldonado A, Catena A. Causal learning in gambling disorder: beyond the illusion of control. J Gambl Stud. (2017) 33:705–17. doi: 10.1007/s10899-016-9634-6.”

The reference for “(50)” was incorrectly written as “50. Ruiz de Lara CM, Navas JF, Soriano-Mas C, Sescousse G, Penelo E. Regional grey matter volume correlates of gambling disorder, gambling-related cognitive distortions, and emotion-driven impulsivity. Int Gambl Stud. (2018) 18:195–216. doi: 10.1080/14459795.2018.14 48427.” It should be “50. Ruiz de Lara CM, Navas JF, Soriano-Mas C, Sescousse G, Perales JC. Regional grey matter volume correlates of gambling disorder, gambling-related cognitive distortions, and emotion-driven impulsivity. Int Gambl Stud. (2018) 18:195–216. doi: 10.1080/14459795.2018.14 48427.”

Additionally, in the published article, there was an error in affiliation “11”. Instead of “Ciber de Salud Mental (CIBERSAM), Instituto Salud Carlos III, Madrid, Spain”, it should be “Department of Psychology, University of Alcalá, Alcalá de Henares, Spain”

The authors apologize for these errors and state that they do not change the scientific conclusions of the article in any way. The original article has been updated.

